# Comparative effectiveness of high-dose versus standard-dose influenza vaccines in nursing home residents aged 65 and older in France: a nationwide cohort study on the French health data system from the 2022–2023 season

**DOI:** 10.1093/ageing/afag238

**Published:** 2026-08-23

**Authors:** Helene Bricout, Marie-Cecile Levant, Pascal Crépey, Gaëtan Gavazzi, Jacques Gaillat, Marine Dufournet, Nada Assi, Benjamin Grenier, Fanny Raguideau, Camille Salamand, Anne Mosnier, Laurence Watier, Odile Launay, Matthew Loiacono

**Affiliations:** Medical Department, Sanofi Pasteur SA, Lyon, Auvergne-Rhône-Alpes, France; Medical Department, Sanofi Pasteur SA, Lyon, Auvergne-Rhône-Alpes, France; Université de Rennes EHESP – UMR CNRS 6051 – ARENES, Rennes, Brittany, France; CHU Grenoble Alpes – Service Universitaire de Gériatrie Clinique, Grenoble, Auvergne-Rhône-Alpes, France; Université Grenoble Alpes – Laboratoire T-Raig TIMC-IMAG CNRS 5525, Saint-Martin-d'Hères, Auvergne-Rhône-Alpes, France; Centre Hospitalier Annecy Genevois – Service de Maladies Infectieuses, Épagny Metz-Tessy, Auvergne-Rhône-Alpes, France; Medical Department, Sanofi Pasteur SA, Lyon, Auvergne-Rhône-Alpes, France; Heva – Biostatistics, Lyon, Auvergne-Rhône-Alpes, France; Heva – Epidemiology, 186 Avenue Thiers, Lyon 69006, France; Heva – Epidemiology, Lyon, France; Medical Department, Sanofi Pasteur SA, Lyon, Auvergne-Rhône-Alpes, France; Open Rome, Paris, Île-de-France, France; Institut Pasteur – Epidemiology and Modeling of Bacterial Escape to Antimicrobials, Paris, Île-de-France, France; Hopital Cochin – Maladies infectieuses et tropicales, Paris, Île-de-France, France; I-REIVAC, Paris, France; Université Paris Cité – Inserm CIC 1417, Paris, Île-de-France, France; Medical Department, Sanofi Pasteur SA, Lyon, Auvergne-Rhône-Alpes, France

**Keywords:** high-dose quadrivalent vaccine, standard-dose quadrivalent vaccine, influenza hospitalisations, ≥ 65 years, nursing home, older people

## Abstract

**Background:**

Older adults in nursing homes face high influenza-related morbidity and mortality due to immunosenescence and comorbidities, which may reduce vaccine effectiveness. High-dose (HD) influenza vaccine, containing four times more antigen than standard-dose (SD) formulations, aim to compensate for this impaired immune response.

**Objectives:**

To compare the Relative Vaccine Effectiveness (rVE) of HD versus SD influenza vaccines against influenza-related hospitalisations (primary), and hospitalisations due to pneumonia or combined influenza/pneumonia (secondary) in nursing home residents aged ≥65 years.

**Study Design:**

Nationwide retrospective cohort (French SNDS). Residents vaccinated between 1 September 2022, and 31 March 2023, were followed until 30 June 2023, discharge or death. Facilities with in-house pharmacies were excluded.

**Exposures/Outcomes:**

HD vs. SD influenza vaccine (pharmacy dispensing codes). Hospitalisations identified by ICD-10 discharge codes.

**Methods:**

HD and SD recipients were matched 1:1 using propensity scores with exact matching on sex, age group, region and vaccination week. Incidence rate ratios (IRR) were estimated using Poisson/negative binomial models. rVE = (1–IRR) × 100.

**Results:**

Among 315 239 residents (123 720 HD; 191 519 SD), 76 858 pairs were matched (mean age 88 years; 75% women). Influenza hospitalisation rates were 302 vs. 438 per 100 000 person-years, corresponding to an rVE of 30.8% (95% CI: 11.8–45.7; *P* = .0029). No significant rVE was observed for pneumonia or combined outcomes.

**Conclusions and Relevance:**

HD vaccine was associated with a significant reduction in influenza-related hospitalisations in French nursing home residents, supporting preferential HD use in this high-risk population. Residual confounding cannot be excluded, and vaccine safety was not assessed in the present analysis.

## Key Points

- Nationwide cohort study using SNDS compared high-dose (HD) versus standard-dose (SD) influenza vaccine effectiveness.
- HD influenza vaccine was associated with a significant reduction in influenza-related hospitalisations in French nursing homes.
- Relative vaccine effectiveness for influenza hospitalisations was 30.8% (95% CI: 11.8–45.7; *P* = .0029).
- Findings support preferential use of high-dose influenza vaccines in institutionalised older populations.

## Introduction

Influenza is a viral illness responsible for an estimated 1 billion cases globally, including 3–5 million severe cases and 290 000–650 000 death [1, 2]. In France, from the 2011–2012 to the 2021–2022 seasons, seasonal influenza resulted in over one million community-based medical consultations, in an excess of 20 000 hospitalisations, and 9000 deaths per season [3]. Adults aged 65 years and above and residents of nursing homes, experience the highest burden of severe outcomes [4–7]. In France, seasonal influenza vaccination is recommended for vulnerable individuals, including those aged 65 and above, those with comorbidities aged 6 months-65 years, residents of nursing homes and pregnant women [8]. The estimated coverage rate among nursing home residents was 87% for the 2022–2023 season. During the 2022–2023 season, which lasted 19 weeks, 313 influenza clusters were reported in French in nursing homes. This number rose to 531 clusters (2023–2024) and 1490 (2024–2025) [9–11].

A key driver of this vulnerability is immunosenescence, the age-related decline in immune function characterised by reduced B- and T-cell responses and impaired vaccine-induced immunity [12]. This phenomenon can impair vaccine-induced immunity and may reduce the effectiveness of influenza vaccines [13]. To address this challenge, a high-dose (HD) influenza vaccine containing four times the antigen content of standard-dose (SD) vaccines (60 μg per strain vs. 15 μg) was developed and approved for use in France in 2020 [14–16]. Evidence from randomised controlled trials and real-world studies has demonstrated superior protection of HD vaccine against laboratory-confirmed influenza and its complications, including hospitalisations for cardio-respiratory outcomes [15–18]. In a cluster randomised trial conducted in the United States, HD demonstrated a reduction in hospitalisations for respiratory illness by 12.7% (95%CI: 1.8–22.4) among nursing home residents compared to SD vaccine [19].

In France, HD vaccine was introduced in 2021–2022 and, while not initially prioritised in national guidelines, has been recommended by the French Society for Geriatric Medicine for preferential use over standard-dose formulations in individuals aged 65 years and older, particularly nursing home residents [20, 21]. We previously reported the nationwide relative vaccine effectiveness (rVE) of the HD versus SD vaccines among community-dwelling individuals aged 65 and over with a 23.3% (95%CI: 8.4–35.8) and 27.4% (19.8–34.3) reduction in influenza hospitalisations during the 2021–2022 and a 2022–2023 seasons [22, 23]. We present the exploratory analysis of the rVE of the HD versus SD vaccines against hospitalisations due to influenza, pneumonia and combined influenza/pneumonia in nursing home residents aged 65 and above during the 2022–2023 season in France.

## Methods

### Study design and data sources

The design of the study is already described elsewhere for community-dwellers [22, 23]. Briefly, this observational cohort study, based on the National Health Data System (Système National Des Données de Santé; SNDS) from the French National Health Insurance system, was designed to describe the characteristics of individuals who received a seasonal influenza vaccine in nursing homes between 1 September 2022, and 31 March 2023, and to assess the rVE of HD vs. SD. The SNDS encompasses anonymous, individual-level data for all healthcare claims for more than 99% of the population residing in France, close to 65 million people [24–28]. Nursing homes admission and discharge date can be identified through the Resid’Ehpad database integrated into the SNDS. Medication consumption at individuals’ level is only available for nursing homes without an in-house Pharmacy (around 77.5% of total nursing homes in France, i.e. 5690 facilities and a theoretical number of 469 369 residents in 2023) [29]. The nursing home unit was unavailable, making impossible to conduct a cluster-level analysis. Furthermore, vaccine choice was likely made at the facility level rather than at the individual resident level, representing a potential source of confounding that could not be fully addressed within the SNDS framework and is discussed as a limitation.

### Study population and study period

All individuals aged ≥65 years, living in nursing homes and with an influenza vaccine dispensed between 1 September 2022, and 31 March 2023 (official end of the French influenza vaccination campaign) were included. Individuals who experienced a hospitalisation for the outcomes of interest between the start of the 2022–2023 influenza season (1 September 2022) and 14 days after vaccine dispensing were excluded. Individuals were followed-up from vaccine dispensing (index date) until 30 June 2023, nursing home discharge, medico-social housing admission or death, whichever came first. Hospitalisations for the outcomes of interest were not treated as censoring events; participants remained in follow-up after a hospitalisation event.

### Variables of interest

#### Exposure

We used pharmacy dispensing records as a proxy for influenza vaccination (Supplementary Methods 1). Vaccine type was classified using medication codes (Supplementary Table S1).

#### Outcomes

The outcomes of interest were the total count of hospitalisations of at least one night due to influenza, pneumonia and influenza and/or pneumonia (Supplementary Table S2). A new event was defined as any admission after discharge from a preceding stay. Outcomes were identified based on the International Classification of Diseases, 10th Revision (ICD-10) discharge diagnosis code and were recorded from 14 days after the index date (start of vaccine protection [30]) until the end of the follow-up period.

#### Covariates

A fixed 5-year pre-index date period was used to capture baseline demographics, comorbidities/medical history and previous treatments and vaccinations (Supplementary Table S3) [31].

### Statistical methods

#### Patient matching

To reduce the bias due to confounding factors, individuals with HD were matched to individuals with SD using a 1:1 ratio propensity score (PS) with an exact constraint on sex, age groups (65–74/75–84/85 years and over), geographical region of residence and week of dispensing of the vaccine. The PS was estimated by logistic regression, which included socio-demographic variables, proxies for health behaviours and comorbidities. The balance between covariates was evaluated using standardised mean differences, with a maximum accepted difference of 10% (Supplementary Methods 2).

#### Main analysis

In the main analysis, we reported endpoints based on stays identified with a primary ICD-10 discharge code, as it should be the main reason for hospitalisation [32]. Hospitalisations with a discharge diagnosis code associated with COVID-19 were excluded.

To ascertain the association between vaccination with HD or SD and hospitalisation for the outcomes of interest, Poisson models, negative binomial models and their zero-inflated counterparts were employed to calculate incidence rate ratios (IRR) with corresponding 95% confidence intervals (95%CI) [33]. The model with the lowest Akaike information criterion was selected [34] (Supplementary Methods 3 & Tables S8–S9). The models included an offset for the log of the follow-up time (starting from day 15 post vaccination), which permitted the calculation of a rate model. The rVE was calculated using the formula ((1–IRR) × 100), with the corresponding 95% CI obtained through the Taylor series variance approximation [35, 36]. *P*-values <.05 were considered as statistically significant and no adjustment for multiplicity was done.

#### Sensitivity analyses

Sensitivity analyses were conducted to assess the robustness of the findings in the main analysis. Given that the PMSI (*Programme de médicalisation des systèmes d’information*) hospital administrative database is maintained for reimbursement purposes, the selection of coding in PMSI could be influenced by the level of severity of the outcome and associated with it by the level of reimbursement that can be claimed by the hospital. We thus used both primary ICD-10 discharge diagnosis codes and non-primary diagnosis codes to identify the study outcomes as a first sensitivity analysis.

Given the high prevalence of SARS-CoV-2 during the 2022–2023 influenza season and the robust surveillance measures in place, we examined individuals with outcomes of interest who also exhibited a diagnosis of SARS-CoV-2 infection (ICD-10 code) in either primary or non-primary diagnosis codes.

To increase the statistical power and maximise the number of HD that would be matched, we also calculated rVE results with a matching ratio varying from 1:1 to 1:4 as a third sensitivity analysis. rVE models were weighted by the proportion of HD recipients matched in each ratio (1:1 to 1:4), scaled to the mean of the matching weights distribution.

Finally, to assess the potential extent of residual bias due to unmeasured confounding, negative control outcomes (NCO) were also analysed; these outcomes shared the same potential sources of bias as the primary outcome but were not plausibly related to the exposure of interest. The effect of HD vs. SD on hospitalisations unrelated to influenza, its complications or influenza vaccination was examined (i.e. urinary tract infection [UTI], cataract surgery or erysipelas) (Supplementary Table S2) [37].

The statistical analyses were performed with SAS Software, Version 9.4 (SAS Institute Inc., Cary, NC, USA).

## Results

**Figure 1:**
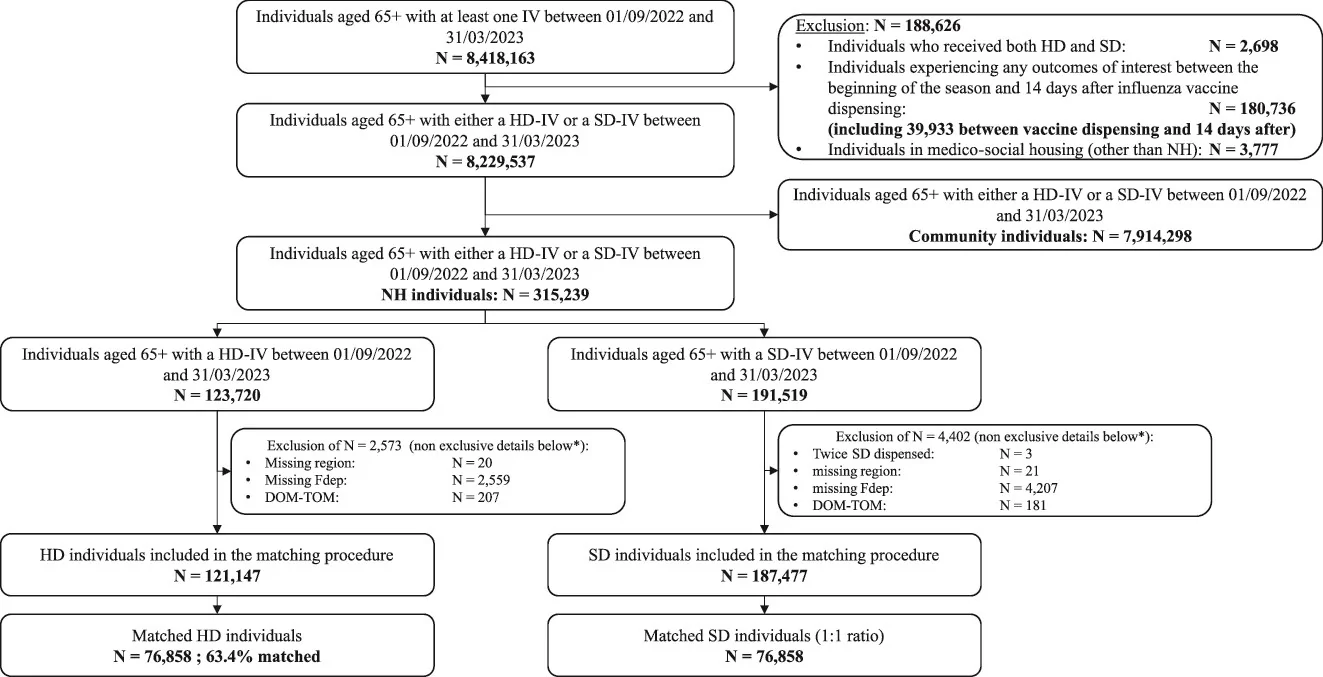
Study population selection process. Individuals with HD were matched to individuals with SD using a 1:1 ratio propensity score (PS) with an exact constraint on sex, age groups (65–74, 75–84, 85 and over), geographical region of residence and week of dispensing of the vaccine. The PS included socio-demographic variables, proxies for health behaviours and comorbidities. DOM-TOM: French overseas territories; HD: High-dose; FDep: French deprivation index; IV: Influenza vaccine; NH: Nursing home; SD: Standard-dose.

A total of 8 418 163 individuals aged 65 and over were identified as having received at least one influenza vaccine between 1 September 2022, and 31 March 2023, in France. A total of 315 239 nursing home residents vaccinated against influenza were identified, comprising 123 720 HD recipients and 191 519 SD recipients. The 1:1 matched cohorts yielded 76 858 HD recipients (representing 63.4% of the initial number of HD recipients) who were matched with 76 858 SD recipients (Figure 1). The remaining 36.6% of HD recipients could not be matched primarily because insufficient SD recipients were available under the exact matching constraints (sex, age group, region and vaccination week).

Individuals’ characteristics were similar after matching (Table 1). The mean age of the subjects was 87.8 (7.7) years in the HD group and 88.0 (7.8) years in the SD group, and 75.3% were women in both groups. Individuals’ characteristics prior to matching are presented in Supplementary Table S4. The most prevalent comorbidities and medical history were cardiovascular diseases (43.9% in HD vs. 43.0% in SD), dementia (41.9% vs. 42.6%) and undernourishment or a history of undernourishment (37.0% vs. 36.6%). The matching performances for the 1:1 ratio are illustrated in Supplementary Figures S1 and S2 and variable ratio matching characteristics and performances in Supplementary Tables S5–S6 and Figure S3.

**Table 1:** Individuals’ characteristics at inclusion (matched cohorts). COPD: Chronic obstructive pulmonary disease; HD: High-dose; SD: Standard-dose.

Characteristics	HD	SD
Number of individuals	76 858	76 858
Age, years		
Mean (SD)	87.76 (7.7)	88.02 (7.8)
Median (IQR)	89.00 (9.0)	89.00 (9.0)
65–70 years, n (%)	2 679 (3.5)	2 507 (3.3)
71–75 years, n (%)	4 107 (5.3)	4 176 (5.4)
76–80 years, n (%)	6 167 (8.0)	5 925 (7.7)
81–85 years, n (%)	11 600 (15.1)	11 945 (15.5)
86–90 years, n (%)	20 566 (26.8)	19 177 (25.0)
91–95 years, n (%)	21 245 (27.6)	21 285 (27.7)
96–100 years, n (%)	9 146 (11.9)	10 230 (13.3)
> 100 years, n (%)	1 348 (1.8)	1 613 (2.1)
Women, n (%)	57 832 (75.3)	57 832 (75.3)
Reasons for end of follow-up, n (%)		
Admission into medico-social housing (other than NH)	18 (0.0)	14 (0.0)
Exit from NH	1 818 (2.4)	2 043 (2.7)
Death	12 333 (16.1)	12 316 (16.0)
End of follow-up	62 689 (81.6)	62 485 (81.3)
Health care seeking behaviours proxy		
All-cause hospitalisation in the past 12 months, mean number per person (SD)	0.15 (0.0)	0.13 (0.0)
Influenza vaccination during the previous season, n (%)	61 839 (80.5)	66 846 (87.0)
COVID-19 vaccination at index date, n (%)	73 927 (96.2)	74 091 (96.4)
Pneumococcal vaccination in the previous 5 years, n (%)	14 325 (18.6)	13 231 (17.2)
Comorbidities, n (%)		
Diabetes	13 139 (17.1)	13 233 (17.2)
Obesity and/or history of obesity surgery	5 958 (7.8)	6 129 (8.0)
Undernourishment or history of undernourishment	28 427 (37.0)	28 105 (36.6)
COPD/Asthma	8 168 (10.6)	8 257 (10.7)
Dementia	32 172 (41.9)	32 768 (42.6)
Cardiovascular diseases	33 715 (43.9)	33 036 (43.0)
Immunocompromised subjects	11 964 (15.6)	11 441 (14.9)
Chronic liver disease	1 328 (1.7)	1 120 (1.5)
Terminal chronic kidney failure	221 (0.3)	214 (0.3)
Number of comorbidities, n (%)		
None	13 392 (17.4)	13 502 (17.6)
1	27 727 (36.1)	27 690 (36.0)
2	20 982 (27.3)	21 112 (27.5)
3	9 728 (12.7)	9 602 (12.5)
4	3 569 (4.6)	3 502 (4.6)
5	1 094 (1.4)	1 101 (1.4)
6 or more	366 (0.5)	349 (0.5)

Standard differences showed good balance for all variables included in the matching procedure (i.e. Absolute value of std. diff <0.1) except for previous influenza vaccination (adjusted for in the models). Characteristics are described for individuals included in the rate models, after exclusion of those with an outcome event or death/loss-to-follow-up during the 14-day post-vaccination lag period.

Influenza-related hospitalisation rates for the main analysis (Primary diagnosis excluding hospitalisations with COVID-19) were 302 (253–360) per 100 000 person-years (PY) in HD recipients and 438 (379–507) per 100 000 PY in SD recipients, which translated into a rVE (95% CI) of 30.8 [11.8–45.7] (*P*-value = .0029) (Table 2 and number of hospitalisation in Supplementary Table S7).

**Table 2:** Relative vaccine effectiveness (rVE [95%CI]) and sensitivity analysis against influenza hospitalisations.

Influenza hospitalisation rate per 100 000 PY (95%CI)		IRR (95%CI)	rVE (95%CI)	*P*-value
Main analysis: Primary diagnosis excluding hospitalisations with COVID-19				
HD	302 (253–360)	**0.69 [0.54–0.88]**	**30.8 [11.8–45.7]**	**.0029**
SD	438 (379–507)			
Sensitivity analysis: Primary or non-primary discharge codes, excluding hospitalisations with COVID-19				
HD	482 (420–554)	**0.73 [0.60–0.89]**	**27.2 [11.4–40.3]**	**.0016**
SD	660 (586–743)			
Sensitivity analysis: Primary diagnosis including hospitalisations with COVID-19				
HD	307 (258–366)	**0.69 [0.54–0.88]**	**30.8 [12.0–45.6]**	**.0027**
SD	446 (385–515)			
Sensitivity analysis: Primary or non-primary discharge codes, including hospitalisations with COVID-19				
HD	504 (440–578)	**0.73 [0.60–0.89]**	**26.9 [11.3–39.8]**	**.0015**
SD	687 (611–772)			

PY: Person-years, IRR: Incidence Rate Ratio, 95% CI: 95% Confidence interval, rVE: Relative Vaccine Effectiveness, HD: High-Dose, SD: Standard Dose. For both IRR and rVE, the SD group is the reference group. Results in bold are statistically significant.

Including hospitalisations with COVID-19 diagnosis induced a rVE (95%CI) of 30.8 [12.0–45.6] (*P*-value = .0027). When using a broader definition considering influenza coded in non-primary discharge position, a rVE (95%CI) of 27.2 [11.4–40.3] (*P*-value = .0016) of HD vs SD was observed. The inclusion of hospitalisations with COVID-19 translated into a rVE (95%CI) of 26.9 [11.3–39.8] (*P*-value = .0015) (Table 2). The results of the variable matching ratio yielded a comparable effect size, which is available in Supplementary Table S10.

No significant difference in relative vaccine effectiveness was observed against pneumonia and pneumonia/influenza hospitalisations (Tables 3 and 4). The incidence of pneumonia and P/I-related hospitalisations was higher in recipients of HD compared to those receiving SD, when analysed in the cohort matched with a variable matching ratio (Supplementary Tables S11 and S12).

**Table 3:** Relative vaccine effectiveness (rVE [95%CI]) and sensitivity analysis against pneumonia hospitalisations.

Pneumonia hospitalisation rate per 100 000 PY (95%CI)		IRR (95%CI)	rVE (95%CI)	*P*-value
Main analysis: Primary diagnosis excluding hospitalisations with COVID-19				
HD	3 771 (3 588–3 964)	1.06 [0.98–1.15]	−6.4 [−15.2–1.8]	.1292
SD	3 550 (3 373–3 737)			
Sensitivity analysis: Primary or non-primary discharge codes, excluding hospitalisations with COVID-19				
HD	6 280 (6 042–6 527)	1.01 [0.95–1.08]	−1.1 [−7.6–5.1]	.7440
SD	6 207 (5 970–6 452)			
Sensitivity analysis: Primary diagnosis including hospitalisations with COVID-19				
HD	3 832 (3 647-4 026)	1.07 [0.99–1.16]	−6.9 [−15.7–1.3]	.1000
SD	3 592 (3 413-3 780)			
Sensitivity analysis: Primary or non-primary discharge codes, including hospitalisations with COVID-19				
HD	6 867 (6 618-7 123)	1.01 [0.95–1.07]	−0.8 [−7.1–5.2]	.8019
SD	6 806 (6 558-7 063)			

PY: Person-years, IRR: Incidence Rate Ratio, 95%CI: 95% Confidence interval, rVE: Relative Vaccine Effectiveness, HD: High-Dose, SD: Standard Dose. For both IRR and rVE, the SD group is the reference group. Results in bold are statistically significant.

**Table 4:** Relative vaccine effectiveness (rVE [95%CI]) and sensitivity analysis against pneumonia/influenza hospitalisations.

	P/I hospitalisation rate per 100 000 PY (95%CI)	IRR (95%CI)	rVE (95%CI)	*P*-value
Main analysis: Primary diagnosis excluding hospitalisations with COVID-19				
HD	4 073 (3 882–4 273)	1.02 [0.95–1.10]	−2.30 [−10.4–5.2]	.5580
SD	3 988 (3 800–4 186)			
Sensitivity analysis: Primary or non-primary discharge codes, excluding hospitalisations with COVID-19				
HD	6 687 (6 441–6 942)	0.99 [0.93–1.05]	1.3 [−4.9–7.1]	.6731
SD	6 762 (6 515–7 018)			
Sensitivity analysis: Primary diagnosis including hospitalisations with COVID-19				
HD	4 139 (3 947–4 340)	1.03 [0.95–1.11]	−2.7 [−10.8–4.7]	.4845
SD	4 037 (3 847–4 236)			
Sensitivity analysis: Primary or non-primary discharge codes, including hospitalisations with COVID-19				
HD	7 371 (7 113–7 639)	0.98 [0.93–1.04]	1.8 [−4.2–7.4]	.5574
SD	7 492 (7 232–7 762)			

PY: Person-years, IRR: Incidence Rate Ratio, 95%CI: 95% Confidence interval, rVE: Relative Vaccine Effectiveness, HD: High-Dose, SD: Standard Dose. For both IRR and rVE, the SD group is the reference group. Results in bold are significant.

The NCO yielded IRR (95%CI) values of 1.06 (0.98–1.15), 1.05 (0.94–1.18) and 1.00 (0.83–1.22) for UTI, cataract surgery and erysipelas hospitalisations, respectively.

## Discussion

During the 2022–2023 influenza season in France, HD in nursing home residents reduced influenza-related hospitalisations by 30.8% (95%CI, 11.8–45.7) compared to SD. Results remained consistent across the range of sensitive analyses conducted. This is in line with a previous research conducted in France during the 2021–2022 influenza season in community-dwelling populations, which reported a rVE of 23.3% (95%CI, 8.4–35.8) [22]. Additionally, our findings align with a cluster randomised trial showing a 12.7% (95%CI, 1.8–22.4) reduction in respiratory illness-related hospitalisations [19]. Notably, our study population was older (mean age of 88.0 years in our cohort vs. 83.6 years in Gravenstein *et al.*) and had lower comorbidity rates (diabetes (17% vs. 34%; COPD/asthma 10.6% vs. 20%)) [19]. Results for pertaining to less specific endpoints, including pneumonia and P/I hospitalisation, were non-significant regardless of outcome definitions. Nonetheless, the effect size reported here against influenza-related hospitalisation is clinically meaningful: in a population where hospitalisation usually translates into either severe disability or death following discharge [5, 7], a 30.8% reduction could translate into 260 avoided hospitalisations, fewer complications and reduced burden on families and healthcare systems.

A major strength of this study is its large, nationwide coverage made possible by the access to the SNDS database. This enabled the study to capture most of the HD vaccine doses distributed in France during the 2022–2023 season. We employed published algorithms to identify most of the individuals’ characteristics, comorbidities and medical history. Increased use of PCR testing for influenza since the COVID-19 pandemic [38, 39] likely improved specificity of influenza coding in hospital records and reduced reliance on broader codes potentially explaining the non-significant results for non-specific endpoints compared to the cluster randomised setting [19]. These strengths contribute to the validity and reliability of our findings regarding influenza hospitalisations, as laboratory-confirmed influenza hospitalisation is the most specific endpoint for evaluating vaccine effectiveness [40–42]. The specificity of this endpoint is crucial in our observational study, given the potential for residual confounding and the high SARS-CoV-2 circulation during the study period. Moreover, while unmeasured confounding cannot be ruled out in an observational study, the NCO analyses yielded non-significant IRR regardless of the outcome, suggesting minimal residual bias.

In this setting, vaccine exposure was not comparable to that observed in community. Indeed, it is a reasonable assumption that a specific nursing home decided to use exclusively HD or SD to vaccinate their residents. As mentioned, the variable indicating the nursing home unit was unavailable, making impossible to conduct a cluster-level analysis, which might have been more appropriate in this context. The analysis was affected by the additional consideration that influenza circulation would likely occur in a cluster effect for nursing homes. Consequently, rVE analyses between HD and SD could not account for comparable influenza circulation between different nursing homes and other disparities at the geographical and unit-specific practices levels (including different vaccine coverage rates for healthcare professionals). This bias was limited by including the department of residence in the matching procedure.

Moreover, our study was limited to residents of nursing homes without in-house pharmacies, because in facilities with such pharmacies, individual vaccine deliveries cannot be tracked through the SNDS claims data. According to the 2019 EHPA study by the French Ministry of Health’s DREES, these nursing homes represent up to 77.5% of all such facilities in France, encompassing an estimated 469 369 residents. In our study, we identified 315 239 individuals who received an influenza vaccine. This corresponds to a theoretical vaccine coverage rate of 67.2%, based on the EHPA population [29]. This estimate is lower than the 87% influenza vaccine coverage reported by *Santé publique France* in a survey of 108 000 residents, which likely included facilities with in-house pharmacies where vaccine coverage may be higher [9]. Consequently, the total number of vaccinated nursing home residents aged 65 and older captured in our dataset is likely underestimated.

According to EHPA data, patient severity, dependency (GIR scale) and in-hospital mortality are broadly similar across facility types [29, 43] supporting generalizability of our findings to the overall nursing home population.

Two thirds (63.4%) of HD recipients were successfully matched to a SD recipient. Analogous pre- and post-matching characteristics make selection bias due to unmatched individuals unlikely. Matching constraints (exact matching variables: sex, age groups, region and vaccination week) prevented the planned 1:4 ratio. A 1:1 ratio was retained for interpretive clarity, with variable ratio matching confirming consistent results.

The limitations related to the use of administrative databases for research purposes also applied to our study. Indeed, we may not have captured all relevant health characteristics of the study individuals. This limits the ability of our matching techniques to adjust for confounding, as without robust estimators of frailty or level of severity for the comorbidity, it is difficult to adjust for these confounding factors, which may lead to an underestimation of vaccine effectiveness. Of note, because the analysis matches HD recipients to SD recipients, the estimated rVE represents the average treatment effect in the treated. These results are therefore most applicable to nursing home residents who received HD vaccination, and may not generalise to all nursing home residents, including those for whom HD was not selected. Other unmeasured confounders missing from administrative databases could have impacted our findings if they were unbalanced between HD and SD recipients. Similarly, the outcome definitions used relied on ICD-10 codes within hospital stays as the database does not include laboratory results (such as influenza or COVID-19 tests results). It can also include hospital-acquired influenza irrespective of SD or HD recipients. Conversely, it does not include patients with influenza who were not hospitalised because deemed too severe. Additionally, mortality during follow-up represents a competing risk for hospitalisation in this population. Future analyses using competing-risk frameworks could further refine these estimates. Furthermore, the 14-day lag period applied to the outcomes means that any very early effect (or differential reactogenicity) of HD versus SD in the first two weeks post-vaccination is not captured by the main analysis. Both numerator (events) and denominator (person-time) correctly exclude this period, but this remains a limitation in terms of generalisability to the earliest post-vaccination window. Furthermore, the exclusion of individuals with a hospitalisation for the outcomes of interest during the pre-vaccination period or lag window (180 736/8 418 163 [2.1%]) means our study population represents a slightly healthier subgroup of nursing home residents. This may limit generalisability of findings to the broader nursing home population. The present analysis focused on effectiveness outcomes and did not assess safety events associated with HD versus SD vaccines. Although existing evidence from clinical trials does not show a clinically significant increase in serious adverse events with HD formulations. Further investigations could also rely on pragmatic trial designs, to confirm these findings and assess broader outcomes (functional decline, quality of life).

## Conclusion

Our study shows that among nursing home residents in France during the 2022–2023 influenza season, HD was associated with a 30.8% (95% CI: 11.8–45.7) reduction in influenza-related hospitalisations, as compared to those who received SD. These findings corroborate the recommendations set forth by the French Society for Geriatric Medicine and the French NITAG, which has been favouring HD over SD in adults 65 years old and older including nursing home residents [20, 21].

## Supplementary Material

aa-25-3177-File003_afag238

## Research Data Transparency and Availability

The data supporting the study findings are part of the National health data system (SNDS, Système national des Données de Santé) and are available from the HDH (Health Data Hub https://www.health-data-hub.fr/). Restrictions apply to the availability of these data containing potentially identifying and sensitive patient information.
