# Supplementary material for: Comparative effectiveness of high-dose versus standard-dose influenza vaccines in nursing home residents aged 65 and older in France: a nationwide cohort study on the French health data system from the 2022–2023 season
Source: Age Ageing. 2026 Aug 23;55(8):afag238. doi: 10.1093/ageing/afag238 (PMC13499703; doi:10.1093/ageing/afag238)
Supplement: Supplementary file 1 [file aa-25-3177-file003_afag238.docx]

**Comparative Effectiveness of High-Dose versus Standard-Dose Influenza Vaccines in Nursing Home Residents Aged 65 and Older in France: A Nationwide Cohort Study on the French Health Data System from the 2022-2023 Season.**

**Supplementary materials**

**Supplementary methods**	**2**

Supplementary Methods 1: Data sources	2

Supplementary Methods 2: Propensity score matching	2

Supplementary Methods 3: Model selection	2

Table S1. Medication codes for SD-IV and HD-IV	3

Table S2. ICD-10 discharge codes for hospitalisations	4

Table S3. Codes used to identify comorbidities	5

**Supplementary results**	**7**

Table S4. Characteristics before matching	7

Figure S1. Propensity-score distribution	9

Figure S2. SMDs — 1:1 fixed matching ratio	10

Figure S3. SMDs — variable matching ratio	11

Table S5. Distribution of matched SD individuals	12

Table S6. Characteristics of variable-ratio matched cohorts	12

Table S7. Events during follow-up	14

Table S8. Regression-model selection — fixed matching ratio	15

Table S9. Regression-model selection — variable matching ratio	16

Table S10. rVE against influenza hospitalisations	17

Table S11. rVE against pneumonia hospitalisations	18

Table S12. rVE against pneumonia/influenza hospitalisations	19

**Supplementary methods**

**Supplementary Methods 1: Data sources**

In the French health care system, individuals who are eligible for influenza vaccination receive a voucher for their influenza vaccine to be dispensed at a pharmacy and then administered by a pharmacist, general practitioner (GP) or nurse (as GP surgeries in France do not store vaccines themselves). We assumed the date of vaccination (“index date”) was approximated by the date of influenza vaccine pharmacy dispensation.

**Supplementary Methods 2: Propensity score matching**

Propensity score (PS) matching was performed using a greedy matching algorithm without replacement with a caliper value equal to 20% of the variability (standard deviation [STD]) of the logarithm of the propensity score values (0.2*STD(log(PS)), with an exact matching constraint on sex, age group (65 to 75 years of age, 75 to 85 years of age, and over 85 years of age), geographical region and week of vaccine dispensing. Geographical region and dispensing week were included to address temporal and geographical gradients likely associated with access to HD-IV (e.g., supply) and influenza virus exposure (i.e., viral activity and circulation). The propensity score was computed using a logistic regression model with vaccine group as the dependent variable and all relevant covariates as independent variables. These independent variables included: number of GP consultations in the past 12 months, number of all-cause hospitalizations in the past 12 months, number of influenza vaccinations (in the five previous seasons), age (continuous, and in categories [used for the exact constraint]: 65-75 years, 75-85 years, above 85 years), sex, vaccine administration setting (either at pharmacy or not), quintiles of the French social deprivation index (FDep), vaccination status for influenza in the preceding season (HD-IV, SD-IV, not vaccinated), COVID-19 vaccination status (completed, on-going [i.e., partial], unvaccinated), pneumonia vaccination status in the past five years, geographical region, and a diagnosis in the previous five years for diabetes, obesity and/or history of obesity surgery (referred to herein as “obesity”), undernourishment or history of undernourishment (referred to herein as “malnutrition”), chronic respiratory diseases, dementia, myocardial infarction, chronic coronary disease, chronic heart failure, peripheral artery disease (PAD), haematological tumours, solid tumours, solid organ transplant, stem cells transplant, HIV (human immunodeficiency virus), drug-induced immunosuppression, chronic liver diseases and severe renal disease, and number of comorbidities of interest.

The quality of the matching was assessed by computing standardized mean differences (SMD) before and after matching. An absolute value of SMD below the cutoff of 0.1 is indicative of a good balance achieved for the covariates^[1]^. In our analysis, SMD absolute values were all below the 0.1 threshold with the exception of previous influenza vaccination, the estimation models for IRR and rVE conducted on the matched population were thus adjusted for the latter variable implementing thus a double robust approach.

**Supplementary Methods 3: Model Selection**

We compared Poisson, negative binomial (NB), zero-inflated Poisson (ZIP), and zero-inflated negative binomial (ZINB) models and selected the final specification according to the Akaike information criterion (AIC). ZIP models NB models were selected for all main analyses, reflecting the excess of zero counts in that analysis subset; NB models were selected for sensitivity analyses using primary or non-primary discharge codes, accounting for overdispersion in event counts (Supplementary Tables S7–S8). We considered the use of robust sandwich standard errors to address potential within-person correlation across recurrent events. However, as >96% of participants experienced zero events and the median number of hospitalizations among cases was 1 (IQR 1–1) across all outcomes and exposure groups, within-person correlation was deemed empirically negligible (Supplementary Table S. The structured AIC-based model-selection procedure, explicitly comparing specifications that allow for overdispersion and excess zeros, was considered sufficient for this count structure.

Table S1. Medication codes (UCD13 and CIP13) for SD-IV and HD-IV

| Vaccine | UCD13 code | CIP13 code |
| --- | --- | --- |
| Standard-dose QIV |  |  |
| - INFLUVAC TETRA SUSP INJ SER 0,5ML | 3400894338703 | 3400930117712 |
| - VAXIGRIPTETRA SUSP INJ SER 0,5ML | 3400894329657 | 3400928099877 |
|  | 3400894329657 | 3400930067727 |
| High-Dose QIV |  |  |
| - EFLUELDA SUSP INJ SER VACCIN | 3400890004602 | 3400930205372 |
|  |  | 3400930205389 |
|  |  | 3400930205426 |
|  |  | 3400930205402 |
|  |  | 3400930205396 |
|  |  | 3400930205419 |

Table S2. ICD-10 discharge codes for hospitalisations

| Definition | ICD-10 codes |
| --- | --- |
| Influenza hospitalisation | J09 to J11 |
| Pneumonia hospitalisation | J12 to J18 |
| Respiratory hospitalisation | J00 to J06, J09 to J18, J40-J41, J96 |
| COVID-19 hospitalisation | U071, U0710, U0711, U0714, U0715 |
| Urinary tract infection hospitalisation | N410, N412-N413, N418, N419, N10, N110, N12, N136, N300, N309, T835 |
| Erysipelas hospitalisation | A46 |
| Cataract hospitalisation | BFGA427, BFGA004, BFPA002, BFGA368, BFGA008, BFGA002, BFGA003, BFGA010, BFGA006, GFGA009 |

Table S3 – ICD-10 codes or references to the codes used to identify comorbidities

| Comorbidity | Codes or references |
| --- | --- |
| Diabetes | Rachas A, Gastaldi-Menager C, Denis P, Barthelemy P, Constantinou P, Drouin J, et al. The Economic Burden of Disease in France From the National Health Insurance Perspective: The Healthcare Expenditures and Conditions Mapping Used to Prepare the French Social Security Funding Act and the Public Health Act. Med Care. 2022;60(9):655-64. |
| Obesity and/or history of obesity surgery | HFCA001, HFCC003, HFFA001, HFFA011, HFFC004, HFFC018, HFGC900, HFKA001, HFKA002, HFKC001, HFLC900, HFLE002, HFMA009, HFMA010, HFMA011, HFMC006, HFMC007, HFMC008, HGCA009, HGCC027, E66 |
| Undernourishment/or history of undernourishment | E43, E44, E46 |
| Chronic respiratory diseases | Rachas A, Gastaldi-Menager C, Denis P, Barthelemy P, Constantinou P, Drouin J, et al. The Economic Burden of Disease in France From the National Health Insurance Perspective: The Healthcare Expenditures and Conditions Mapping Used to Prepare the French Social Security Funding Act and the Public Health Act. Med Care. 2022;60(9):655-64. |
| Dementia | Rachas A, Gastaldi-Menager C, Denis P, Barthelemy P, Constantinou P, Drouin J, et al. The Economic Burden of Disease in France From the National Health Insurance Perspective: The Healthcare Expenditures and Conditions Mapping Used to Prepare the French Social Security Funding Act and the Public Health Act. Med Care. 2022;60(9):655-64. |
| Cardiovascular diseases (including myocardial infarction (MI), chronic coronary disease, chronic heart failure, Peripheral Artery disease (PAD)) | Rachas A, Gastaldi-Menager C, Denis P, Barthelemy P, Constantinou P, Drouin J, et al. The Economic Burden of Disease in France From the National Health Insurance Perspective: The Healthcare Expenditures and Conditions Mapping Used to Prepare the French Social Security Funding Act and the Public Health Act. Med Care. 2022;60(9):655-64. |
| Immunocompromised individuals (including cancer patients (solid and hematological tumours), organ/stem cells transplanted patients, HIV patients, and chronic autoimmune or inflammatory diseases treated with immunosuppressive or biologic drugs) | Rachas A, Gastaldi-Menager C, Denis P, Barthelemy P, Constantinou P, Drouin J, et al. The Economic Burden of Disease in France From the National Health Insurance Perspective: The Healthcare Expenditures and Conditions Mapping Used to Prepare the French Social Security Funding Act and the Public Health Act. Med Care. 2022;60(9):655-64.  And  Wyplosz B, Fernandes J, Goussiaume G, Moïsi J, Lortet-Tieulent J, Vainchtock A, et al. Adults at risk of pneumococcal disease in France. Infect Dis Now. 2021 Nov;51(8):661–6. |
| Chronic liver disease | Rachas A, Gastaldi-Menager C, Denis P, Barthelemy P, Constantinou P, Drouin J, et al. The Economic Burden of Disease in France From the National Health Insurance Perspective: The Healthcare Expenditures and Conditions Mapping Used to Prepare the French Social Security Funding Act and the Public Health Act. Med Care. 2022;60(9):655-64. |
| Terminal chronic kidney failure | Rachas A, Gastaldi-Menager C, Denis P, Barthelemy P, Constantinou P, Drouin J, et al. The Economic Burden of Disease in France From the National Health Insurance Perspective: The Healthcare Expenditures and Conditions Mapping Used to Prepare the French Social Security Funding Act and the Public Health Act. Med Care. 2022;60(9):655-64. |

**Supplementary results**

Table S4. Individuals’ characteristics at inclusion (cohorts before matching)

| Characteristics | HD | SD |
| --- | --- | --- |
| Number of individuals | 123,720 | 191,519 |
| Age, years |  |  |
| Mean (SD) | 87.95 (7.8) | 87.66 (8.0) |
| Median (IQR) | 89.00 (9.0) | 89.00 (10.0) |
| 65-70 years, n (%) | 4,277 (3.5) | 7,592 (4.0) |
| 71-75 years, n (%) | 6,657 (5.4) | 11,272 (5.9) |
| 76-80 years, n (%) | 9,760 (7.9) | 15,556 (8.1) |
| 81-85 years, n (%) | 17,928 (14.5) | 27,858 (14.6) |
| 86-90 years, n (%) | 32,104 (26.0) | 49,317 (25.8) |
| 91-95 years, n (%) | 34,725 (28.1) | 52,575 (27.5) |
| 96-100 years, n (%) | 15,831 (12.8) | 23,681 (12.4) |
| >100 years, n (%) | 2,438 (2.0) | 3,668 (1.9) |
| Women, n (%) | 93,989 (76.0) | 144,155 (75.3) |
| Reasons for end of follow-up, n (%) |  |  |
| Admission into medico-social housing (other than NH) | 30 (0.0) | 37 (0.0) |
| Exit from NH | 2,605 (2.1) | 4,890 (2.6) |
| Death | 20,211 (16.3) | 29,870 (15.6) |
| End of follow-up | 100,874 (81.5) | 156,722 (81.8) |
| Health care seeking behaviours proxy |  |  |
| All-cause hospitalisation in the past 12 months, mean (SD) | 0.13 (0.9) | 0.13 (0.8) |
| Influenza vaccination during the previous season, n (%) | 107,962 (87.3) | 164,590 (85.9) |
| COVID-19 vaccinated*, n (%) | 119,457 (96.6) | 184,245 (96.2) |
| Pneumococcal vaccination in the previous 5 years, n (%) | 25,455 (20.6) | 31,769 (16.6) |
| Comorbidities, n (%) |  |  |
| Diabetes | 20,901 (16.9) | 32,934 (17.2) |
| Obesity and/or history of obesity surgery | 9,575 (7.7) | 14,778 (7.7) |
| Undernourishment or history of undernourishment | 44,128 (35.7) | 67,385 (35.2) |
| COPD/Asthma | 12,910 (10.4) | 20,449 (10.7) |
| Dementia | 51,860 (41.9) | 77,475 (40.5) |
| Cardiovascular diseases | 52,351 (42.3) | 81,709 (42.7) |
| Immunocompromised subjects | 18,358 (14.8) | 28,203 (14.7) |
| Chronic liver disease | 1,900 (1.5) | 2,809 (1.5) |
| Terminal chronic kidney failure | 333 (0.3) | 499 (0.3) |
| Number of comorbidities, n (%) |  |  |
| None | 22,321 (18.0) | 35,841 (18.7) |
| 1 | 45,384 (36.7) | 69,838 (36.5) |
| 2 | 33,386 (27.0) | 51,491 (26.9) |
| 3 | 15,156 (12.3) | 22,929 (12.0) |
| 4 | 5,361 (4.3) | 8,072 (4.2) |
| 5 | 1,588 (1.3) | 2,520 (1.3) |
| 6 or more | 524 (0.4) | 828 (0.4) |
| Region of residence at index date, n (%) |  |  |
| Missing | 20 | 21 |
| Guadeloupe | 0 (0.0) | 2 (0.0) |
| Martinique | 0 (0.0) | 1 (0.0) |
| Other DROM | 4 (0.0) | 1 (0.0) |
| Ile-de-France | 16,088 (13.0) | 22,284 (11.6) |
| Centre-Val de Loire | 5,071 (4.1) | 8,044 (4.2) |
| Bourgogne-Franche-Comté | 4,753 (3.8) | 10,251 (5.4) |
| Normandie | 6,399 (5.2) | 8,769 (4.6) |
| Hauts-de-France | 12,130 (9.8) | 10,889 (5.7) |
| Grand Est | 11,320 (9.2) | 12,711 (6.6) |
| Pays de la Loire | 9,067 (7.3) | 18,173 (9.5) |
| Bretagne | 7,097 (5.7) | 14,732 (7.7) |
| Nouvelle Aquitaine | 12,699 (10.3) | 20,826 (10.9) |
| Occitanie | 10,683 (8.6) | 23,021 (12.0) |
| Auvergne-Rhône-Alpes | 15,410 (12.5) | 24,242 (12.7) |
| Provence-Alpes-Côte d'Azur | 12,731 (10.3) | 16,700 (8.7) |
| Corse | 45 (0.0) | 675 (0.4) |
| DROM unprecised | 198 (0.2) | 169 (0.1) |
| COM unprecised | 5 (0.0) | 8 (0.0) |
| Week of vaccine administration, n (%) |  |  |
| Week 1: 35 (1 Sept) | 1 (0.0) | 0 (0.0) |
| Week 2: 36 (5 Sept) | 0 (0.0) | 1 (0.0) |
| Week 3: 37 (12 Sept) | 0 (0.0) | 3 (0.0) |
| Week 4: 38 (19 Sept) | 1 (0.0) | 2 (0.0) |
| Week 5: 39 (26 Sept) | 5 (0.0) | 9 (0.0) |
| Week 6: 40 (3 Oct) | 58 (0.0) | 53 (0.0) |
| Week 7: 41 (10 Oct) | 175 (0.1) | 243 (0.1) |
| Week 8: 42 (17 Oct) | 27,900 (22.6) | 35,370 (18.5) |
| Week 9: 43 (24 Oct) | 22,210 (18.0) | 28,608 (14.9) |
| Week 10: 44 (31 Oct) | 14,903 (12.1) | 23,449 (12.2) |
| Week 11: 45 (7 Nov) | 15,284 (12.4) | 24,671 (12.9) |
| Week 12: 46 (14 Nov) | 15,331 (12.4) | 27,315 (14.3) |
| Week 13: 47 (21 Nov) | 9,447 (7.6) | 17,446 (9.1) |
| Week 14: 48 (28 Nov) | 5,740 (4.6) | 11,075 (5.8) |
| Week 15: 49 (5 Dec) | 3,302 (2.8) | 5,997 (3.1) |
| Week 16: 50 (12 Dec) | 2,502 (2.0) | 5,022 (2.6) |
| Week 17: 51 (19 Dec) | 1,848 (1.5) | 3,003 (1.6) |
| Week 18: 52 (26 Dec) | 1,562 (1.3) | 2,678 (1.4) |
| Week 19: 1 (2 Jan) | 1,025 (0.8) | 2,124 (1.1) |
| Week 20: 2 (9 Jan) | 657 (0.5) | 1,483 (0.8) |
| Week 21: 3 (16 Jan) | 702 (0.6) | 1,293 (0.7) |
| Week 22: 4 (23 Jan) | 500 (0.4) | 869 (0.5) |
| Week 23: 5 (30 Jan) | 150 (0.1) | 502 (0.3) |
| Week 24: 6 (6 Feb) | 50 (0.0) | 80 (0.0) |
| Week 25: 7 (13 Feb) | 110 (0.1) | 56 (0.0) |
| Week 26: 8 (20 Feb) | 147 (0.1) | 53 (0.0) |
| Week 27: 9 (27 Feb) | 99 (0.1) | 47 (0.0) |
| Week 28: 10 (6 Mar) | 5 (0.0) | 22 (0.0) |
| Week 29: 11 (13 Mar) | 3 (0.0) | 17 (0.0) |
| Week 30: 12 (20 Mar) | 2 (0.0) | 16 (0.0) |
| Week 31: 13 (27 Mar) | 1 (0.0) | 12 (0.0) |
| DROM/COM: Overseas Territories | | |

Figure S1: Distribution of propensity score in both high-dose and standard-dose groups

Figure S2. Standardized mean differences (SMD) of the variables included in the propensity score (1:1 fixed matching ratio)

Figure S3. Standardized mean differences (SMD) of the variables included in the propensity score (Variable matching ratio)

Table S5. Distribution of the number of SD matched to HD individuals (Variable ratio matched cohorts)

| No. of SD individuals part of a quartet, triplet, pair or unary (%) | SD |
| --- | --- |
| SD-QIV part of a control quartet | 44,940 (25.4) |
| SD-QIV part of a control triplet | 56,778 (32.1) |
| SD-QIV part of a control pair | 57,338 (32.4) |
| One SD-QIV matched | 18,028 (10.2) |
| Total number of SD matched within the variable matching ratio | 177,084 (100.0) |

Table S6. Individuals’ characteristics at inclusion (Variable ratio matched cohorts)

| Characteristics | HD | SD |
| --- | --- | --- |
| Number of individuals | 76,858 | 76,858 |
| Age, years |  |  |
| Mean (SD) | 87.76 (0.0) | 87.92 (0.0) |
| Median (IQR) | 88.56 (9.5) | 88.76 (9.2) |
| 65-70 years, n (%) | 2,679 (3.5) | 2,604 (3.4) |
| 71-75 years, n (%) | 4,107 (5.3) | 4,190 (5.5) |
| 76-80 years, n (%) | 6,167 (8.0) | 6,124 (8.0) |
| 81-85 years, n (%) | 11,600 (15.1) | 11,635 (15.1) |
| 86-90 years, n (%) | 20,566 (26.8) | 19,528 (25.4) |
| 91-95 years, n (%) | 21,245 (27.6) | 21,334 (27.8) |
| 96-100 years, n (%) | 9,146 (11.9) | 9,904 (12.9) |
| >100 years, n (%) | 1,348 (1.8) | 1,539 (2.0) |
| Women, n (%) | 57,832 (75.3) | 57,832 (75.3) |
| Reasons for end of follow-up, n (%) |  |  |
| Admission into medico-social housing (other than NH) | 18 (0.0) | 17 (0.0) |
| Exit from NH | 1,818 (2.4) | 1,994 (2.6) |
| Death | 12,333 (16.1) | 12,248 (15.9) |
| End of follow-up | 62,689 (81.6) | 62,599 (81.5) |
| Health care seeking behaviours proxy |  |  |
| All-cause hospitalisation in the past 12 months, mean (SD) | 0.15 (0.0) | 0.13 (0.0) |
| Influenza vaccination during the previous season, n (%) | 61,839 (80.5) | 66,846 (87.0) |
| COVID-19 vaccinated*, n (%) | 73,927 (96.2) | 74,091 (96.4) |
| Pneumococcal vaccination in the previous 5 years, n (%) | 14,325 (18.6) | 13,231 (17.2) |
| Comorbidities, n (%) |  |  |
| Diabetes | 13,139 (17.1) | 13,271 (17.3) |
| Obesity and/or history of obesity surgery | 5,958 (7.8) | 6,092 (7.9) |
| Undernourishment or history of undernourishment | 28,427 (37.0) | 27,867 (36.3) |
| COPD/Asthma | 8,168 (10.6) | 8,271 (10.8) |
| Dementia | 32,172 (41.9) | 32,025 (41.7) |
| Cardiovascular diseases | 33,715 (43.9) | 33,043 (43.0) |
| Immunocompromised subjects | 11,964 (15.6) | 11,422 (14.9) |
| Chronic liver disease | 1,328 (1.7) | 1,105 (1.4) |
| Terminal chronic kidney failure | 221 (0.3) | 203 (0.3) |
| Number of comorbidities, n (%) |  |  |
| None | 13,392 (17.4) | 13,749 (17.9) |
| 1 | 27,727 (36.1) | 27,781 (36.2) |
| 2 | 20,982 (27.3) | 21,020 (27.4) |
| 3 | 9,728 (12.7) | 9,484 (12.3) |
| 4 | 3,569 (4.6) | 3,398 (4.4) |
| 5 | 1,094 (1.4) | 1,077 (1.4) |
| 6 or more | 366 (0.5) | 348 (0.5) |
| Region of residence at index date, n (%) |  |  |
| Missing | 0 (0.0) | 0 (0.0) |
| Guadeloupe | 0 (0.0) | 0 (0.0) |
| Martinique | 0 (0.0) | 0 (0.0) |
| Other DROMs | 0 (0.0) | 0 (0.0) |
| Ile-de-France | 11,233 (14.6) | 11,233 (14.6) |
| Centre-Val de Loire | 3,005 (3.9) | 3,005 (3.9) |
| Bourgogne-Franche-Comté | 3,300 (4.3) | 3,300 (4.3) |
| Normandie | 2,939 (3.8) | 2,939 (3.8) |
| Hauts-de-France | 6,410 (8.3) | 6,410 (8.3) |
| Grand Est | 6,257 (8.1) | 6,257 (8.1) |
| Pays de la Loire | 5,583 (7.3) | 5,583 (7.3) |
| Bretagne | 4,723 (6.2) | 4,723 (6.2) |
| Nouvelle Aquitaine | 8,019 (10.4) | 8,019 (10.4) |
| Occitanie | 7,390 (9.6) | 7,390 (9.6) |
| Auvergne-Rhône-Alpes | 10,323 (13.4) | 10,323 (13.4) |
| Provence-Alpes-Côte d'Azur and Corse | 7,676 (10.0) | 7,676 (10.0) |
| Corse | 0 (0.0) | 0 (0.0) |
| DROM unprecised | 0 (0.0) | 0 (0.0) |
| COM unprecised | 0 (0.0) | 0 (0.0) |
| Week of vaccine administration, n (%) |  |  |
| Week 1: 35 (1 Sept) | 0 (0.0) | 0 (0.0) |
| Week 2: 36 (5 Sept) | 0 (0.0) | 0 (0.0) |
| Week 3: 37 (12 Sept) | 0 (0.0) | 0 (0.0) |
| Week 4: 38 (19 Sept) | 0 (0.0) | 0 (0.0) |
| Week 5: 39 (26 Sept) | 0 (0.0) | 0 (0.0) |
| Week 6: 40 (3 Oct) | 6 (0.0) | 6 (0.0) |
| Week 7: 41 (10 Oct) | 46 (0.1) | 46 (0.1) |
| Week 8: 42 (17 Oct) | 16,022 (20.9) | 16,022 (20.9) |
| Week 9: 43 (24 Oct) | 12,975 (16.9) | 12,975 (16.9) |
| Week 10: 44 (31 Oct) | 9,476 (12.3) | 9,476 (12.3) |
| Week 11: 45 (7 Nov) | 9,700 (12.6) | 9,700 (12.6) |
| Week 12: 46 (14 Nov) | 10,122 (13.2) | 10,122 (13.2) |
| Week 13: 47 (21 Nov) | 6,252 (8.1) | 6,252 (8.1) |
| Week 14: 48 (28 Nov) | 4,193 (5.5) | 4,193 (5.5) |
| Week 15: 49 (5 Dec) | 2,428 (3.2) | 2,428 (3.2) |
| Week 16: 50 (12 Dec) | 1,883 (2.5) | 1,883 (2.5) |
| Week 17: 51 (19 Dec) | 1,113 (1.5) | 1,113 (1.5) |
| Week 18: 52 (26 Dec) | 986 (1.3) | 986 (1.3) |
| Week 19: 1 (2 Jan) | 627 (0.8) | 627 (0.8) |
| Week 20: 2 (9 Jan) | 397 (0.5) | 397 (0.5) |
| Week 21: 3 (16 Jan) | 382 (0.5) | 382 (0.5) |
| Week 22: 4 (23 Jan) | 135 (0.2) | 135 (0.2) |
| Week 23: 5 (30 Jan) | 63 (0.1) | 63 (0.1) |
| Week 24: 6 (6 Feb) | 9 (0.0) | 9 (0.0) |
| Week 25: 7 (13 Feb) | 7 (0.0) | 7 (0.0) |
| Week 26: 8 (20 Feb) | 11 (0.0) | 11 (0.0) |
| Week 27: 9 (27 Feb) | 25 (0.0) | 25 (0.0) |
| Week 28: 10 (6 Mar) | 0 (0.0) | 0 (0.0) |
| Week 29: 11 (13 Mar) | 0 (0.0) | 0 (0.0) |
| Week 30: 12 (20 Mar) | 0 (0.0) | 0 (0.0) |
| Week 31: 13 (27 Mar) | 0 (0.0) | 0 (0.0) |
| Standard differences showed good balance for all variables included in the matching procedure (i.e., Absolute value of std. diff < 0.1) except for previous influenza vaccination (adjusted for in the models). DROM/COM/TOM: Overseas Territories | | |

Table S7. Number of events over the follow-up (Fixed ratio matched cohorts)

|  | Outcomes, N (%) otherwise specified | | HD (N=76,858) | | SD (N=76,858) | |
| --- | --- | --- | --- | --- | --- | --- |
|  | Influenza hospitalization | |  | |  | |
|  | Presence | | 199 (0.26) | | 267 (0.35) | |
|  | Mean number (SD) | | 0.00 (0.05) | | 0.00 (0.06) | |
|  | Median number (Q1-Q3) | | 0 (0–0) | | 0 (0–0) | |
|  | Min-Max number | | 0 – 2 | | 0 – 3 | |
|  | Mean number (SD) among cases | | 1.04 (0.20) | | 1.06 (0.25) | |
|  | Median number (Q1-Q3) among cases | | 1 (1–1) | | 1 (1–1) | |
|  | Min-Max number among cases | | 1 – 2 | | 1 – 3 | |
|  | Pneumonia hospitalization | |  | |  | |
|  | Presence | | 2,539 (3.30) | | 2,504 (3.26) | |
|  | Mean number (SD) | | 0.04 (0.21) | | 0.04 (0.21) | |
|  | Median number (Q1-Q3) | | 0 (0–0) | | 0 (0–0) | |
|  | Min-Max number | | 0 – 5 | | 0 – 6 | |
|  | Mean number (SD) among cases | | 1.11 (0.01) | | 1.12 (0.01) | |
|  | Median number (Q1-Q3) among cases | | 1 (1–1) | | 1 (1–1) | |
|  | Min-Max number among cases | | 1 – 5 | | 1 – 6 | |
| Pneumonia and/or Influenza hospitalization | |  | |  | |  |
|  | Presence | | 2,694 (3.51) | | 2,708 (3.52) | |
|  | Mean number (SD) | | 0.04 (0.22) | | 0.04 (0.22) | |
|  | Median number (Q1-Q3) | | 0 (0–0) | | 0 (0–0) | |
|  | Min-Max number | | 0 – 5 | | 0 – 6 | |
|  | Mean number (SD) among cases | | 1.12 (0.37) | | 1.14 (0.41) | |
|  | Median number (Q1-Q3) among cases | | 1 (1–1) | | 1 (1–1) | |
|  | Min-Max number among cases | | 1 – 5 | | 1 – 6 | |

Table S8 – Choice of regression model to compute incidence rate ratios with corresponding Akaike Information Criteria (AIC) for the 1:1 fixed matching ratio. The model with the bold AIC value was selected for further analysis. ZIP: Zero-inflated Poisson / ZINB: Zero-inflated Negative Binomial.

| **Hospitalisation outcomes** | **Distribution** | **Main analysis: Primary diagnosis excluding hospitalisations with COVID-19** | **Main analysis: Primary diagnosis including hospitalisations with COVID-19** | **Main analysis: Primary or non-primary discharge codes, excluding hospitalisations with COVID-19** | **Main analysis: Primary or non-primary discharge codes, including hospitalisations with COVID-19** |
| --- | --- | --- | --- | --- | --- |
| Influenza | Poisson | 4579.5943 | 4644.8155 | 6691.3586 | 6940.9447 |
|  | ZIP | **4507.9192** | **4573.9377** | 6573.5553 | 6811.2326 |
|  | Negative Binomial | 4508.6900 | 4574.6905 | **6573.3605** | **6810.8282** |
|  | ZINB | 4509.9208 | 4575.9393 | 6575.3282 | 6812.8289 |
| Pneumonia | Poisson | 31548.2617 | 31913.3167 | 48411.5172 | 52173.7015 |
|  | ZIP | **30801.4138** | **31157.3694** | 47025.4043 | 50666.5365 |
|  | Negative Binomial | 30806.5013 | 31163.1055 | **47011.2076** | **50646.9027** |
|  | ZINB | 30803.2768 | 31159.2964 | 47012.2220 | 50648.3100 |
| Pneumonia and/or influenza | Poisson | 34123.7662 | 34524.1763 | 51382.6780 | 55879.2209 |
|  | ZIP | **33328.3213** | **33716.4523** | 49944.7339 | 53957.5444 |
|  | Negative Binomial | 33335.6745 | 33724.7282 | **49929.5508** | **53932.1234** |
|  | ZINB | 33330.3122 | 33718.4530 | 49930.4900 | 53933.3377 |

Table S9 – Choice of regression model to compute incidence rate ratios with corresponding Akaike Information Criteria (AIC) for the variable matching ratio. The model with the bold AIC value was selected for further analysis.. ZIP: Zero-inflated Poisson / ZINB: Zero-inflated Negative Binomial.

| **Hospitalisation outcomes** | **Distribution** | **Main analysis: Primary diagnosis excluding hospitalisations with COVID-19** | **Sensitivity analysis: Primary diagnosis including hospitalisations with COVID-19** | **Sensitivity analysis: Primary or non-primary discharge codes, excluding hospitalisations with COVID-19** | **Sensitivity analysis: Primary or non-primary discharge codes, including hospitalisations with COVID-19** |
| --- | --- | --- | --- | --- | --- |
| Influenza | Poisson | 7829.4855 | 7959.8647 | 11399.7612 | 11790.8943 |
|  | ZIP | **7689.3922** | **7821.5249** | **11134.2183** | **11505.1622** |
|  | Negative Binomial | 8293.4691 | 8422.6752 | 11957.4553 | 12317.1492 |
|  | ZINB | 8115.7570 | 8246.8477 | 11638.2239 | 11989.6272 |
| Pneumonia | Poisson | 51881.0472 | 52509.9597 | 79865.8811 | 86205.3727 |
|  | ZIP | 50608.8664 | 51223.8882 | 77548.1724 | 83692.5763 |
|  | Negative Binomial | 48790.2447 | 49404.9428 | 75646.7995 | 81650.9798 |
|  | ZINB | **48738.3718** | **49350.9223** | **75512.5324** | **81511.1198** |
| Pneumonia and/or influenza | Poisson | 56334.9994 | 57032.3049 | 85075.6580 | 92536.8557 |
|  | ZIP | 54981.0046 | 55666.9933 | 82614.8800 | 89331.6443 |
|  | Negative Binomial | 53480.0587 | 54164.2473 | 81045.9295 | 87633.5241 |
|  | ZINB | **53429.0161** | **54110.9312** | **80915.3883** | **87482.9301** |

Table S10. Relative vaccine effectiveness (rVE [95% CI]) and sensitivity analyses against influenza hospitalisations (Variable matching ratio. PY: Person-years, IRR: Incidence Rate Ratio, 95% CI: 95% Confidence interval, rVE: Relative Vaccine Effectiveness, HD: High-Dose, SD: Standard Dose, IV: Influenza Vaccine. For both IRR and rVE, the SD group is the reference group. Results in bold are significant.

| Influenza hospitalization rate per 100,000 PY (95% CI) | | IRR (95% CI) | rVE (95% CI) | p-value |
| --- | --- | --- | --- | --- |
| Main analysis: Primary diagnosis excluding hospitalisations with COVID-19 | |  |  |  |
| HD-QIV | 302 (253-360) | **0.65 [0.54;0.78]** | **35.4 [22.1;46.5]** | **<.0001** |
| SD-QIV | 454 (413-499) |  |  |  |
| Sensitivity analysis: Primary or non-primary discharge codes, excluding hospitalisations with COVID-19 | |  |  |  |
| HD-QIV | 482 (420-554) | **0.68 [0.58;0.79]** | **31.9 [20.6;41.6]** | **<.0001** |
| SD-QIV | 683 (632-737) |  |  |  |
| Sensitivity analysis: Primary diagnosis including hospitalisations with COVID-19 | | | | |
| HD-QIV | 307 (258-365) | **0.64 [0.53;0.77]** | **35.7 [22.6;46.6]** | **<.0001** |
| SD-QIV | 463 (421-508) |  |  |  |
| Sensitivity analysis: Primary or non-primary discharge codes, including hospitalisations with COVID-19 | | | | |
| HD-QIV | 504 (440-578) | **0.69 [0.59;0.80]** | **31.2 [20.0;40.9]** | **<.0001** |
| SD-QIV | 704 (652-759) |  |  |  |

Table S11. Relative vaccine effectiveness (rVE [95% CI]) and sensitivity analyses against pneumonia hospitalisations (Variable matching ratio). PY: Person-years, IRR: Incidence Rate Ratio, 95% CI: 95% Confidence interval, rVE: Relative Vaccine Effectiveness, HD: High-Dose, SD: Standard Dose, IV: Influenza Vaccine. For both IRR and rVE, the SD group is the reference group. Results in bold are significant.

| Pneumonia hospitalization rate per 100,000 PY (95% CI) | | IRR (95% CI) | rVE (95% CI) | p-value |
| --- | --- | --- | --- | --- |
| Main analysis: Primary diagnosis excluding hospitalisations with COVID-19 | |  |  |  |
| HD-QIV | 3,771 (3,588-3,964) | **1.12 [1.05;1.20]** | **-12.1 [-20.1;-4.7]** | **0.0011** |
| SD-QIV | 3,353 (3,238-3,471) |  |  |  |
| Sensitivity analysis: Primary or non-primary discharge codes, excluding hospitalisations with COVID-19 | |  |  |  |
| HD-QIV | 6,280 (6,042-6,527) | **1.06 [1.00;1.12]** | **-5.8 [-11.6;-0.3]** | **0.0391** |
| SD-QIV | 5,910 (5,757-6,066) |  |  |  |
| Sensitivity analysis: Primary diagnosis including hospitalisations with COVID-19 | | | | |
| HD-QIV | 3,832 (3,648-4,026) | **1.12 [1.05;1.20]** | **-12.4 [-20.3;-5.0]** | **0.0008** |
| SD-QIV | 3,402 (3,287-3,522) |  |  |  |
| Sensitivity analysis: Primary or non-primary discharge codes, including hospitalisations with COVID-19 | | | | |
| HD-QIV | 6,867 (6,618-7,125) | 1.05 [1.00;1.11] | -5.2 [-10.8;0.1] | 0.0525 |
| SD-QIV | 6,498 (6,338-6,663) |  |  |  |

Table S12. Relative vaccine effectiveness (rVE [95% CI]) and sensitivity analyses against pneumonia/influenza (P/I) hospitalisations (Variable matching ratio). PY: Person-years, IRR: Incidence Rate Ratio, 95% CI: 95% Confidence interval, rVE: Relative Vaccine Effectiveness, HD: High-Dose, SD: Standard Dose, IV: Influenza Vaccine. For both IRR and rVE, the SD group is the reference group. Results in bold are significant.

| P/I hospitalization rate per 100,000 PY (95% CI) | | IRR (95% CI) | rVE (95% CI) | p-value |
| --- | --- | --- | --- | --- |
| Main analysis: Primary diagnosis excluding hospitalisations with COVID-19 | |  |  |  |
| HD-QIV | 4,073 (3,882-4,273) | 1.06 [1.00;1.14] | -6.5 [-13.7;0.3] | 0.0603 |
| SD-QIV | 3,807 (3,685-3,933) |  |  |  |
| Sensitivity analysis: Primary or non-primary discharge codes, excluding hospitalisations with COVID-19 | |  |  |  |
| HD-QIV | 6,687 (6,441-6,942) | 1.02 [0.97;1.08] | -2.2 [-7.6;3.0] | 0.4143 |
| SD-QIV | 6,502 (6,342-6,667) |  |  |  |
| Sensitivity analysis: Primary diagnosis including hospitalisations with COVID-19 | | | | |
| HD-QIV | 4,139 (3,947-4,340) | 1.07 [1.00;1.14] | -6.7 [-13.9;0.0] | 0.0502 |
| SD-QIV | 3,865 (3,742-3,992) |  |  |  |
| Sensitivity analysis: Primary or non-primary discharge codes, including hospitalisations with COVID-19 | | | | |
| HD-QIV | 7,371 (7,113-7,639) | 1.02 [0.97;1.07] | -1.6 [-6.8;3.4] | 0.5362 |
| SD-QIV | 7,202 (7,033-7,375) |  |  |  |

1. Stuart EA, Lee BK, Leacy FP. Prognostic score-based balance measures can be a useful diagnostic for propensity score methods in comparative effectiveness research. J Clin Epidemiol. 2013 Aug;66(8 Suppl):S84-S90.e1.

   Zhang Z, Kim HJ, Lonjon G, Zhu Y. Balance diagnostics after propensity score matching. Ann Transl Med. 2019 Jan;7(1):16.
